# Clinical application of 10 600 and 1540 nm laser therapy in post-burn functional impairment: a case report

**DOI:** 10.1097/RC9.0000000000000572

**Published:** 2026-06-02

**Authors:** Giuseppe Scarcella, Irene Fusco, Anna Sara Gervasi, Francesca Madeddu, Tiziano Zingoni

**Affiliations:** a General Secretary and National Head of Laser Department of ISPLAD, International-Italian Society of Plastic-Regenerative and Oncologic Dermatology, Verona, Italy; b Department of Clinical Research and Practice, El.En. Group, Calenzano, Italy

**Keywords:** 1540 nm, burn, CO_2_ laser, hypertrophic scar

## Abstract

**Introduction:**

Extensive burn injury can result in hypertrophic, retracting scars that, based on their location, can lead to reduced functionality for the patient. This study reports the clinical case of a young man who presented at the clinic with second-degree burn injuries to the chin, hand, and forearm.

**Case presentation:**

The patient complained of pruritus and had difficulty moving his hand, which impacted his professional life. The scars were treated with the sequential emission of two laser wavelengths: 10 600 and 1540 nm. The patient was very satisfied with the outcome and was able to resume his work.

**Discussion:**

Recent histological studies have demonstrated that the sequential emission of the CO_2_ laser at 10 600 and the 1540 nm non-ablative laser extends the thermal effects on the micro-ablative zone, stimulating and enhancing collagen remodeling compared with the use of CO_2_ alone. The sequential emission of these two wavelengths has been proven to be a valid treatment choice for skin remodeling for aesthetic purposes, with shorter healing times and a lesser risk of hypopigmentation.

**Conclusion:**

This study used a combination of ablative and non-ablative lasers to remodel hypertrophic burn scars, achieving improved aesthetics, symptom relief, and restored hand function through the resolution of joint contractures.

## Introduction

A burn is an injury to the skin or other tissues caused by heat, radiation, electricity, friction, or chemicals. It is classified by severity, from first to fourth degree[1].

First-degree burns are superficial and usually need no treatment. Second-degree burns penetrate the dermis, causing pain and fluid secretion; they require dressings and may scar but not surgery. Third-degree burns destroy the dermis and reach fat, causing scars and contraction, and need surgical treatment and infection control. Fourth-degree burns extend to muscle, bone, or tendon and require specialized care, possibly including flaps or amputation[2].

Wound healing is a complex process influenced by burn size, age, health, and infection[3]. It includes an inflammatory phase (removal of dead tissue and growth factor release), a proliferative phase (formation of vessels and connective tissue), and a remodeling phase (collagen and elastin reorganization)^[^1,4^]^.

HIGHLIGHTS

- The human body, in response to an injury, activates the wound-healing process.
- Dysregulation of the wound-healing process leads to scar formation.
- Sequential laser emission of 10 600 and 1540 nm wavelengths can be used for the management of hypertrophic and retracting scars.

Disruptions in healing can cause pathological scars, such as hypertrophic scars[5], which are raised but confined to the wound area and linked to excess collagen III[6].

Although burn incidence is decreasing globally, especially in high-income countries[7], non-fatal burns can significantly impact quality of life. Up to 70% of patients develop hypertrophic scars^[^8,9^]^, leading to contractures, reduced mobility, and aesthetic concerns that may hinder social reintegration[8]. Recommended treatment includes fractional ablative laser, alone or with other therapies[9].

Fractional ablative CO_2_ laser (10 600 nm) creates microscopic ablation zones (DOT) surrounded by thermal areas that stimulate tissue regeneration^[^10,11^]^, improving scar appearance[12]. Combining 10 600 nm with 1540 nm wavelengths enhances thermal effects and collagen production compared to CO_2_ alone^[^13,14^]^. Based on these scientific findings, this study evaluated the use of a dual-wavelength system with a fractional scanning unit for the management of hypertrophic and retracting scars caused by second- and third-degree burn injuries.

This case report has been reported in line with the SCARE checklist[15].

## Case presentation

A 23-year-old male physiotherapist, skin type IV, presented at the clinic (DermoLaser Office, Verona, Italy) with three hypertrophic retracting scars: one beneath the chin (12 × 9 cm), one on the right hand (13 × 12 cm), and one on the left forearm (11 × 3 cm). In December 2023, he sustained second- and third-degree burns due to a backfire while lighting wood with diesel fuel. He was initially treated with skin autografts from his left thigh to the right hand and forearm.

The hypertrophic and retracting scarring outcome had a huge impact on his daily and professional life. The extension of the head and the arm was significantly limited, accompanied by a persistent sensation of pruritus and significant aesthetic discomfort. Moreover, he experienced a loss of functionality and dexterity in his right hand, which precluded him from resuming his work.

The patient was treated with a laser device (DuoGlide, Deka M.E.L.A. Srl, Florence, Italy) that allows the sequential emission of 10 600 and 1540 nm wavelengths, with a scanning unit (μScan DOT) for the fractional emission of the laser.

The treatment consisted of three sessions, at around 30 days apart. The laser emission was in smart-pulse mode, stack 2, with a distance between DOTs (spacing) of 500 µm. The sequence of emission was 10 600 nm (power 16 W, dwell time 1000 µs) followed by the 1540 nm emission (power 3 W, dwell time 5 µs). Immediately after the laser treatment, triamcinolone acetonide 40 mg/ml was applied topically to the treated areas. No adverse effects were observed, and no topical anaesthetic was applied. The pain scale from 1 to 5 (1 = no pain, 2 = slight pain, 3 = medium pain, 4 = severe pain, 5 = extreme pain) revealed that the patient reported a 3.5, which represents a medium but tolerable pain score.

An antibiotic cream, to be applied twice a day, was prescribed for the first week post-treatment. Until the next treatment, solar protection during the day and topical silicone at night were recommended.

Each scar was evaluated with the modified Vancouver Scar Scale (mVSS) before and after the treatments. The mVSS consists of a numerical assessment of four skin characteristics and two indicators for patient subjective symptoms: pliability (range 0–5), vascularity, pigmentation, height (range 0–3), pain, and pruritus (range 0–2)[16].

**Figure 1.**
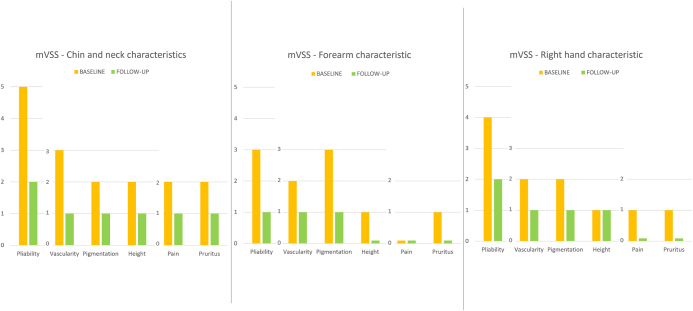
The mVSS score at the baseline and at follow-up, for each scar.

At 2 months’ follow-up, after the last treatment, the total mean score of the mVSS scale was halved, from 12.3 ± 3.2 to 5.0 ± 2.0. The scars appeared less pliable and pigmented, with a reduction in pain and pruritus (Fig. 1).

**Figure 2.**
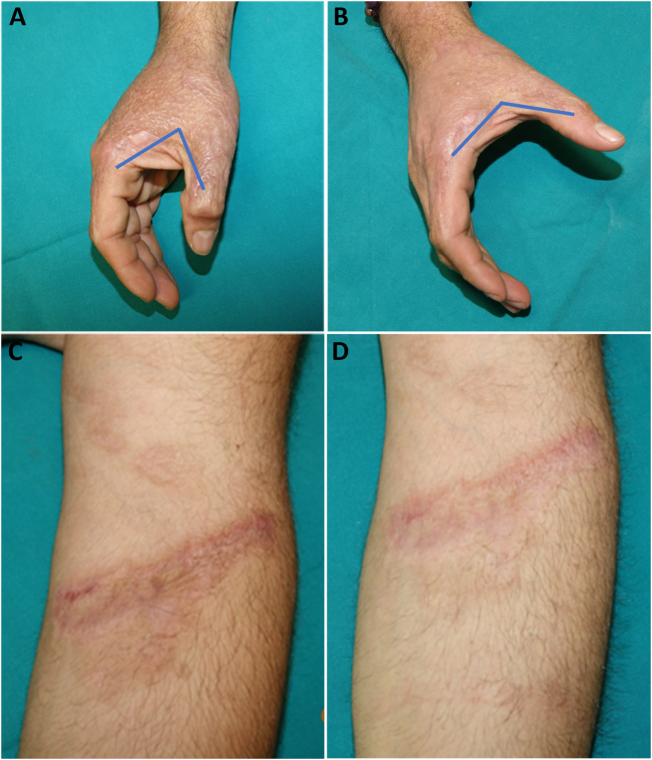
Improvement of patient’s scar before (A) and after (B) the laser sessions. An increase in the abduction angle of the thumb of the right hand was observed (B). Improvement of patient’s scar on forearm area before (C) and after (D) the laser sessions.

**Figure 3.**
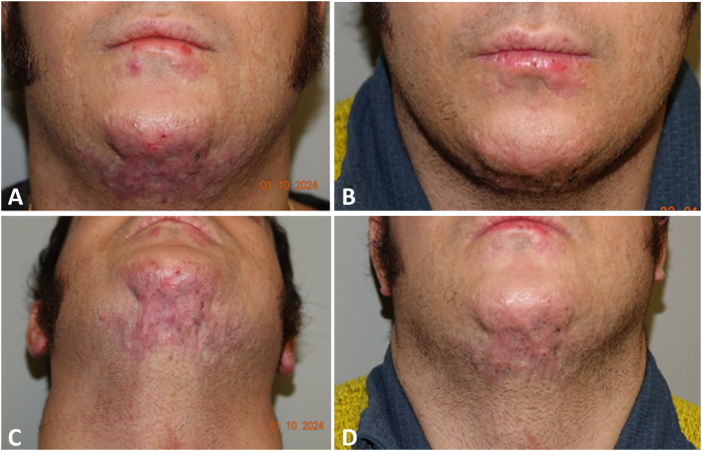
Improvement of patient’s scars on beneath the chin before (A,C) and after (B,D) the laser sessions.

The patient reported an improvement in the scars’ appearance and was very satisfied with the symptomatic and functional outcomes of the treatments (Figs. 2 and 3). Indeed, he substantially regained the extension of the neck, with a notable reduction in the itching sensation. On the hand, the most significant result was obtained, with an increase in the abduction angle of the thumb (Fig. 2, panels A and B). This was a crucial aspect of the restoration of the patient’s manual dexterity and the opportunity to resume his work.

## Discussion

Post-burn scars can impair physical and mental well-being for up to 10 years, causing aesthetic discomfort, itching, sleep disturbances, and reduced daily functioning[17]. They may also lead to joint contractures and functional limitations, with 28% of patients not returning to work, especially in cases of extensive scarring^[^18,19^]^. Rehabilitation is therefore a key component of burn care, aiming to restore function and improve daily activities and social participation^[^1,4^]^. Fractional ablative lasers are currently the first-line treatment for hypertrophic scar contractures, used alone or with other therapies such as topical or intralesional treatments[9]. Laser-based therapies are increasingly studied in burn care, with a growing role in reconstructive and rehabilitative management.

As highlighted in the review by Riva *et al*[20], different laser modalities have shown promising results in improving scar quality, including pliability, thickness, and functional impairment. However, despite the growing body of evidence, these approaches are not yet fully standardized, as treatment protocols, indications, and timing remain heterogeneous across studies.

In this context, the present case report does not introduce a new therapeutic strategy but describes the clinical application and functional outcomes of a combined dual-wavelength approach in routine clinical practice. Recent histological studies show that the sequential emission of 10 600 nm CO_2_ and 1540 nm lasers enhances thermal effects in the micro-ablative zone, promoting collagen remodeling more effectively than CO_2_ alone^[^13,14^]^. This combined approach is supported by evidence of a synergistic interaction; in particular, Nisticò *et al*[13] demonstrated in an *ex vivo* model that the two wavelengths produce additive and complementary effects on dermal remodeling. The 1540 nm non-ablative wavelength extends the thermal diffusion generated by CO_2_ micro-ablative columns, creating a more homogeneous dermal effect and enhancing collagen remodeling without increasing tissue damage. Overall, evidence indicates that this combined approach is not experimental but represents an evolution of established laser-assisted skin remodeling techniques. This case report highlights improvements in scar appearance, along with high patient satisfaction regarding symptomatic relief and functional recovery following combined dual-wavelength laser treatment.

Regarding the treatment protocol (three sessions at 30-day intervals), the schedule adopted in this case report is consistent with both commonly reported clinical practice and the biological timeline of wound healing following fractional laser therapy. Previous studies have shown that fractional CO_2_ laser treatments are typically performed in multiple sessions (commonly 2–5) spaced approximately 4 weeks apart, allowing adequate dermal remodeling between treatments[21]. Clinical evidence also supports this approach, as protocols based on three sessions at 1-month intervals have demonstrated significant improvements in Vancouver Scar Scale scores[22]. This interval is further supported by the physiology of skin repair, since fibroblast activity and collagen remodeling peak within several weeks after laser-induced microthermal injury, making a 4-week interval optimal to balance efficacy and tissue recovery[23]. Therefore, the protocol used in our study reflects current evidence-based practice.

Preclinical evidence from experimental and histological studies, including *in vitro* and *ex vivo* animal models, has extensively investigated the biological effects of fractional CO_2_ laser and combined wavelength approaches. These studies show that laser-induced microthermal zones promote fibroblast proliferation, neocollagenesis, and collagen remodeling. Fractional CO_2_ laser induces controlled thermal injury, triggering inflammation, cell proliferation, and dermal reorganization, supporting repeated treatment sessions[24]. Several preclinical and clinical studies have evaluated the combined use of 10 600 nm CO_2_ and 1540 nm lasers, showing a synergistic effect that extends the thermal coagulation zone without increasing ablation depth and enhances collagen remodeling[25]. *Ex vivo* studies confirm deeper and more homogeneous dermal effects compared to CO_2_ alone[13], while *in vitro* data demonstrate increased fibroblast proliferation and neocollagenesis after 1540 nm exposure[14].

This dual-wavelength approach is also effective for aesthetic skin remodeling, offering faster healing and a lower risk of hypopigmentation^[^26,27^]^. A main limitation of the present study is the relatively short follow-up period.

Future investigations should include longer follow-up to better assess the durability of the clinical outcomes over time. In addition, incorporating independent, blinded evaluators for aesthetic assessment and validated patient-reported outcome measures would provide a more comprehensive evaluation of both the clinical and psychosocial effects of the treatment, in line with previous recommendations in the literature^[^28,29^]^.

## Conclusion

In this study, a combination of ablative and non-ablative lasers was used to treat hypertrophic and retracting burn scars, resulting in improved aesthetic outcomes, symptom reduction, and restored hand function through the resolution of joint contractures.

## Data Availability

The data that support the findings of this study are not publicly available since it contains information that could compromise the privacy of research participants but are available from the corresponding author (I.F.) upon reasonable request.
